# Genomic insights into the serovar prevalence, antimicrobial resistance gene, and genetic diversity of *Salmonella enterica* in Mexico

**DOI:** 10.1371/journal.pone.0323872

**Published:** 2025-05-15

**Authors:** Luis Lozano-Aguirre, Josefina Duran-Bedolla, Juan Téllez-Sosa, Cindy Fabiola Hernández-Pérez, Ismael Hernández-Lucas, Humberto Barrios-Camacho

**Affiliations:** 1 Programa de Genómica Evolutiva, Centro de Ciencias Genómicas, Universidad Nacional Autónoma de México, Cuernavaca, Morelos, México; 2 Instituto Nacional de Salud Pública (INSP), Centro de Investigación Sobre Enfermedades Infecciosas (CISEI), Cuernavaca, Morelos, México; 3 Centro Nacional de Referencia en Inocuidad y Bioseguridad Agroalimentaria del SENASICA, Tecámac, Estado de México, México; 4 Departamento de Microbiología Molecular, Instituto de Biotecnología, Universidad Nacional Autónoma de México, Cuernavaca, Morelos, México; Cornell University, UNITED STATES OF AMERICA

## Abstract

The research aims to provide insights into the sources of contamination, prevalence of common serovars, determination of sequence types, prediction of genes associated with antimicrobial resistance, and phylogenetic analysis to evaluate genetic diversity and correlations between serovars and sequence types in *Salmonella enterica* in Mexico. We analyzed 818 publicly accessible whole-genome sequences from Mexico, which included isolates from diverse sources such as poultry, meat, water, and agricultural environments. We identified fifty-seven serovars, of which 19.8% corresponded to *S*. Infantis, 10.7% to *S*. Anatum, and 6.6% to *S*. Newport, representing the most common serovars. Phylogenetic analysis shows a strong correlation between sequence type and serovar. For example, ST32 for *S*. Infantis and ST64 for *S*. Anatum show this. However, serovars such as S. Newport possessed considerable genomic diversity, suggesting complex contamination pathways. The analysis showed that many isolates have been identified as multidrug-resistant, exhibiting resistance gene profiles for aminoglycosides, β-lactams, fluoroquinolones, sulfonamides, and tetracyclines. The findings emphasize the importance of identifying contamination sources to monitor the dissemination of multidrug-resistant *Salmonella* in regions that have significant antibiotic consumption in agriculture and farming, highlighting its global relevance for food safety and public health.

## Introduction

*Salmonella enterica* is a bacterial pathogen that causes several diseases, including self-limiting gastroenteritis and more severe systemic infections such as typhoid fever. *S. enterica* infections, a primary contributor to global foodborne illnesses, account for millions of cases each year, with estimates between 200 million and over 1 billion infections globally, including around 93 million instances of gastroenteritis and 155,000 fatalities, primarily associated with contaminated food and water sources [1,2].

Approximately 85% of *Salmonella* infections are correlated to contaminated food, especially chicken, meat, and eggs, which create ideal conditions for *Salmonella* proliferation due to their elevated nutritional and water content [3]. Moreover, fruits and vegetables contaminated by animal fecal matter might serve as incubators for *Salmonella* proliferation [4].

In countries with inadequate sanitation protocols, such as Mexico, the incidence of *Salmonella* infections remains notably high. In 2020, Mexico documented 64,778 instances of *Salmonella* infections, translating it a significant public health concern [5]. The issue is compounded by the increasing prevalence of multidrug-resistant (MDR) *Salmonella* strains, which complicates treatment and control efforts. While resistance to antibiotics like β-lactams, aminoglycosides, tetracyclines, macrolides, and quinolones has been reported for several decades, the emergence of resistance to third-generation cephalosporins, fluoroquinolones, and azithromycin has become more noticeable in the past two decades [6,7].

The emergence of resistant strains, particularly in nations with significant agricultural antibiotic usage, has intensified the need for comprehensive surveillance of *Salmonella* serovars and the antimicrobial resistance (AMR) patterns. *S. enterica* exhibits considerable serovar variety, encompassing approximately 2,500 recognized serovars, each differing in pathogenic capability, environmental adaptation, and host specificity [8,9]. Common serovars, including Enteritidis, are frequently recovered from poultry, whereas Typhimurium, Newport and Infantis are more widely detected from different sources including various animals’ species [10,11].

Besides serovar diversity, the genomic characteristics of *S. enterica*, including the presence of antimicrobial resistance genes, offer essential insights into the evolution of resistance mechanisms. Genomic surveillance is essential in regions like Mexico, where rapid changes in agricultural practices and antibiotic usage patterns drive the emergence of MDR *Salmonella* strains [12]. Nonetheless, despite the substantial increase in *Salmonella* genomes available in public databases, initiatives to utilize this data for real-time surveillance of contamination sources and AMR trends remain limited.

In this study, we analyzed 818 *S. enterica* genomes from Mexico, obtained from various sources such as raw meat, fruits, vegetables, water, and soil. The isolates comprise 57 non-typhoidal *Salmonella* serovars, showing the variety of contamination sources in Mexico. Furthermore, numerous isolates possess genes associated with resistance against aminoglycosides, fluoroquinolones, β-lactams, and various other types of antibiotics. Using openly available genomic data, we offer a novel insight into the epidemiology of *Salmonella* in Mexico, specifically emphasizing antibiotic resistance trends and the genetic relationship between serovar and sequence types.

## Materials and methods

Illumina paired-end sequences corresponding to 822 *S. enterica* genomes, sequenced by the Centro Nacional de Referencia de Inocuidad y Bioseguridad Agroalimentaria, a division of the Servicio Nacional de Sanidad, Inocuidad y Calidad Agroalimentaria (SENASICA) institution, were retrieved from the NCBI SRA database in February 2024. The quality of the raw sequencing reads was assessed using FASTQC v0.12.1, and low-quality bases and adapter sequences were removed using Trim Galore v0.6.10. The trimmed reads were then assembled using SPAdes v3.15.5 [13]. To evaluate the quality of the assembly, the CheckM (v1.2.2) program was used, eliminating those genomic assemblies that did not have ≥90% completeness and ≤5% with no evidence of contaminant contigs [14]. Serovar identification was performed using stringMLST from the PubMLST database to assign serovars based on sequence data. Additionally, antigenic formulas (O- and H-antigens) were determined using SeqSero2 (www.denglab.info/SeqSero2), providing molecular confirmation of the serovars [15]. These results were further cross validated using the NCBI Pathogen Detection platform, which confirmed the serovar identifications through its own algorithms.

This process resulted in 818 high-quality *S. enterica* genome assemblies. Functional annotation of these genomes was conducted using Prokka v1.14.6, generating comprehensive gene annotations [16] (S1 Metadata).

### Average nucleotide identity

The average nucleotide identity (ANI) analysis of the 818 genomes was performed using the PYANI program v0.2.12 with the ANIm (Average Nucleotide Identity by MUMmer) alignment method [17]. An ANI heatmap was generated using the *pheatmap* package (v1.0.12) in RStudio, based on the *ANIm_percentage_identity.tab* file.

### Multilocus sequencing typing (MLST) and eBURST analysis

Multilocus sequence typing (MLST) for *Salmonella* was performed using the Achtman scheme, targeting the genes *aroC*, *dnaN*, *hemD*, *hisD*, *purE*, *sucA*, and *thrA*. Sequence types were determined by querying the PubMLST database (https://pubmlst.org/organisms/salmonella-spp) [18]. Clonal complexes (CCs) were identified using the Global Optimal eBURST (goeBURST) algorithm via PHYLOViZ 2.0 software (https://www.phyloviz.net/goeburst). The analysis of clonal complexes was conducted at the level of single-locus variants (SLVs) [19].

### *In Silico* prediction of antibiotic resistance genes

Antibiotic resistance genes (ARGs) were identified using the Resistance Gene Identifier (RGI) v6.0.3, integrated with the Comprehensive Antibiotic Resistance Database (CARD v3.2.9). Both “Strict” and “Perfect” detection criteria were employed to ensure a high level of confidence in the identification of ARGs. The “Perfect” criterion identifies genes with exact matches to known resistance determinants, while the “Strict” criterion allows for minor sequence variation, capturing potential novel variants that may still confer resistance. This dual approach provides a comprehensive prediction of both well-characterized and potentially emerging resistance genes [20].

### Core genome and phylogenetic analysis

To examine the phylogenetic relationships among the *S. enterica* genomes, we performed a core genome analysis using ROARY v3.13.0 [21]. The analysis identified a core genome consisting of 3,252 genes, which were present in 100% of the 818 isolates and used as the basis for phylogenetic inference. The concatenated core genome alignment was used for phylogenomic reconstruction with RAxML v8.2.10, a maximum likelihood-based phylogenetic tool optimized for large datasets [22]. The General Time Reversible (GTR) model with gamma-distributed rate heterogeneity was applied to account for evolutionary rate variation. All analyses, including those performed using ROARY, RAxML, and other tools (e.g., CARD, RGI), were conducted using default settings. Bootstrapping was performed with 100 replicates to assess the statistical support for the branching patterns in the phylogenetic tree.

## Results

### Genetic diversity of *Salmonella enterica* in Mexico

The genomic metadata analysis of 818 *S. enterica* isolates from Mexico reveals their distribution by collection year and sample origin as follows: In **2017**, a total of 87 isolates were recovered from avian caeca (n = 17), bovine lymph nodes (n = 34), ground beef (n = 11), water (n = 11), and other sources (n = 14). In **2018**, 77 isolates were obtained from avian caeca (n = 22), bovine lymph nodes (n = 13), chicken (n = 15), ground beef (n = 19), water (n = 4), and other sources (n = 4). In **2019**, 293 isolates were collected, originating from avian caeca (n = 151), chicken (n = 74), raw chicken (n = 58), and other sources (n = 10). In **2020**, 49 isolates were identified from chicken (n = 16) and raw chicken (n = 33). In **2021**, 144 isolates were retrieved from ground beef (n = 139) and ground pork (n = 5). In **2022**, 168 isolates were recovered from ground beef (n = 36), ground pork (n = 102), and water (n = 30).

The overall distribution of isolates by sample origin was as follows: Avian-caeca 23.2% (190/818), Bovine-lymph-node 5.7% (47/818), Chicken 12.7% (105/818), Ground-beef 25% (205/818), Ground-pork 12.9% (107/818), Raw-chicken 11.1% (91/818), Water 5.5% (45/818), and other sources 3.4% (28/818) (S1 Metadata).

**Fig 1:**
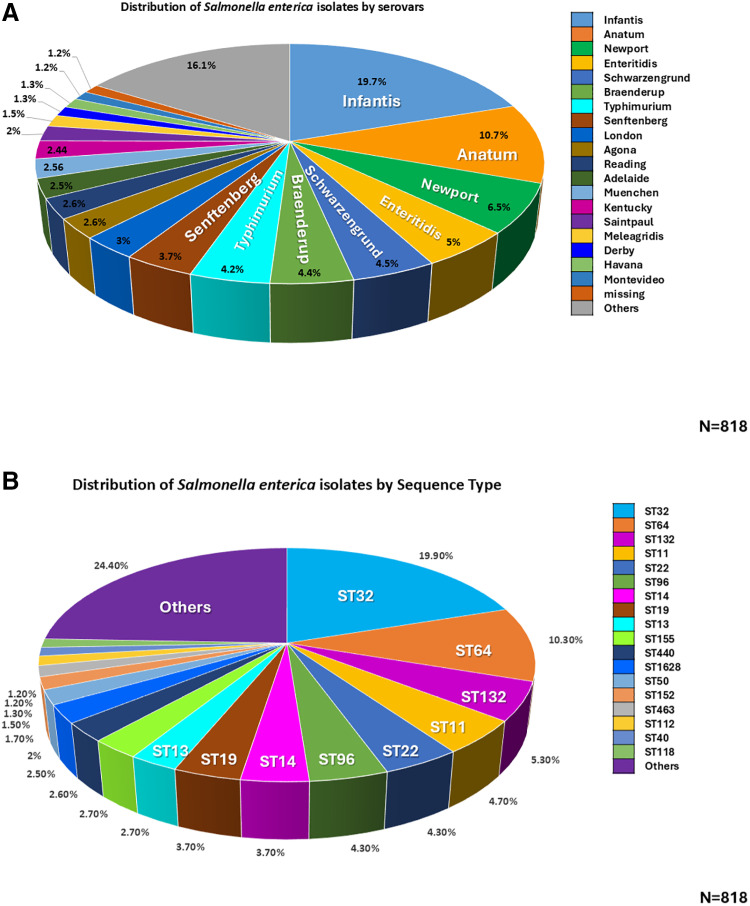
Distribution of *S. enterica* isolates by serovars (A) and sequences type (B) identified from 818 isolates collected in Mexico.

The distribution observed of *S. enterica* collected from the sources mentioned above demonstrates a broad range of serovars within the population. A total of 57 different serovars were identified among the 818 genomes. Among these, the predominant serovars were: Infantis 19.6% (161/818), Anatum 10.7% (88/818), Newport 6.5% (54/818), Enteritidis 5% (41/818), Schwarzengrund 4.5% (37/818), Braenderup 4.4% (36/818), Typhimurium 4.2% (35/818), Senftenberg 3.7% (30/818), London 3% (25/818), Agona 2.6% (22/818), Reading 2.6% (22/818), Adelaide 2.5% (21/818), Muenchen 2.5% (21/818), Kentucky 2.4% (20/818), Saintpaul 2% (17/818), Meleagridis 1.5% (13/818), Havana 1.3% (11/818), Derby 1.3% (11/818), and Montevideo 1.2% (10/818). These 19 main serovars collectively represented 82.5% of the genomes analyzed. In 1.2% (9/818) of the genomes, the specific serovar could not be identified (Fig 1a).

The remaining 38 serovars, account for 16.2% (133/818) of the genomes analyzed, includes uncommon serovars with lower frequency but significant epidemiological relevance, emphasizing the wide-ranging presence of *Salmonella* serovars across contamination sources (S1 Metadata).

The correlation between genomes belonging to a specific serovar was corroborated by ANI analysis, which validated the serovar assignments through a high level of genome sequence identity (S2 Metadata). This approach reinforced the accuracy of serovar identification across the *S. enterica* isolates, supporting consistent serovar clustering within the genomes.

### Temporal epidemiology of the main serovars

To explore the temporal epidemiology of the most prevalent *S. enterica* serovars Infantis, Anatum, and Newport we analyzed the dates of collection of the isolates obtained between 2017 and 2022.

For Infantis serovar, the 161 genomes were obtained from 63 different dates of collection. Most isolates recovered in 2019, with 105 genomes collected in 45 different dates. Smaller numbers of isolates were observed in 2017 (4 isolates, 3 dates), 2018 (7 isolates, 6 dates), 2020 (18 isolates, 4 dates), 2021 (11 isolates, 1 date), and 2022 (16 isolates, 2 dates).

In the Anatum serovar, the 88 genomes were collected on 46 different dates. Most of the isolates were obtained in 2021, with 28 isolates from 9 dates, followed by 19 isolates from 15 dates in 2017. The remaining isolates were distributed across 2018 (10 isolates, 5 dates), 2019 (13 isolates, 11 dates), 2020 (3 isolates, 2 dates), and 2022 (15 isolates, 4 dates).

In the case of *S.* Newport (n = 54), isolates were obtained from 20 different dates across four years. Most of the isolates were collected in 2022, with 27 isolates collected on 9 dates, and in 2021, with 19 isolates during 7 dates. In contrast, less isolates were detected in 2019 (7 isolates, 3 dates) and only one single isolate in 2017.

### *Salmonella* sequence type distribution

The distribution of *S. enterica* isolates by sequence type (ST), provides insight into the genetic diversity within the sampled population. Among the 809 genomes for which serovars were identified, ST assignment was determined using the Achtman scheme. From this analysis 83 different ST were detected. The most prevalent serovar was Infantis with 19.7% (160/809) associated with ST32. The second most frequent serovar was Anatum accounting for 10.2% (83/809) and primarily associated with ST64. Other frequently observed STs included ST132 5.3% (43/809), ST11 4.6% (38/809), ST22 and ST96 4.4% (36/809), ST19 3.8% (31/809), ST14 3.7% (30/809), ST13 2.8% (23/809), ST155 2.7% (22/809), ST440 2.5% (21/809), and ST1628 2.4% (20/809). These 12 STs comprise 67.2% (544/809) of the genomes analyzed (Fig 1b).

Notably, the remaining 71 STs, representing less common identified, accounted for 32.7% (265/809) of the genomes, indicating a substantial number of low-frequency STs and highlighting the genetic diversity within the *S. enterica* population (S1 Metadata). This variation across sequence types suggests multiple contamination sources and pathways within Mexico’s food production and environmental systems, emphasizing the need for broad-spectrum surveillance to track emerging variants and potential resistance trends.

### Correlation between specific serovars and ST distribution

The distribution of *S. enterica* genomes by both serovars and ST reveals a close association between serovars diversity and genetic variation within the analyzed genomes. Of the 57 different serovars identified among the genomes analyzed, 36.8% (21/57) of the serovars were associated with more than one ST, accounting for 76.1% (616/809). Conversely, 63.1% (36/57) of serovars were associated with a single ST, representing 23.8% (193/809) of the genomes analyzed.

Among these 36 serovars exclusively correlated with a single ST (n = 193), the five most prevalent associations were ST14 with Senftenberg (30/193), ST13 with Agona (22/193), ST440 with Adelaide (21/193), ST463 with Meleagridis (13/193), and ST40 with Derby (11/193). Collectively, these five STs constituted 50.2% (97/193) of the genomes with a one-to-one correlation between serovar and ST.

**Fig 2:**
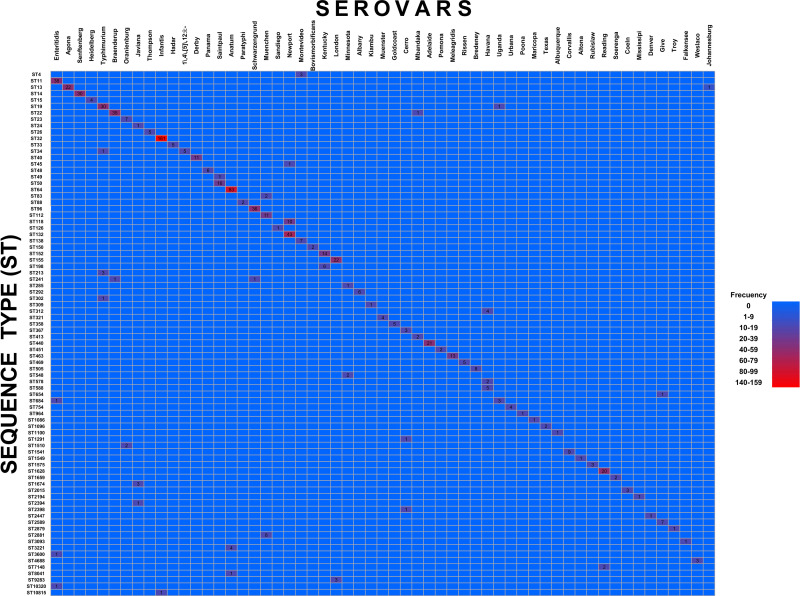
Relationship between serovars and sequence types of *S. enterica.* The heat map shows the frequency of each ST associated with specific serovars.

For serovars associated with multiple STs (n = 616), a predominant ST was identified, demonstrating a consistent correlation between each serovar and a dominant ST (Fig 2). The seven main serovars represented by more than one ST were as follows: Infantis, comprising 26.2% (162/616), was predominantly represented by ST32 99.3%, (161/162) and minimally by ST10815 0.7% (1/162); Anatum, accounting for 14.2% (88/616), was mainly associated with ST64 94.3% (83/88), followed by ST3221 4.5% (4/88) and ST8041 1.1% (1/88); Newport, at 8.7% (54/616), was represented primarily by ST132 79.6% (43/54), with additional associations to ST118 18.5% (10/54) and ST45 1.8% (1/54); Enteritidis, at 6.6% (41/616), was primarily linked to ST11 92.6% (38/41), with minor associations to ST684 2.4% (1/41), ST3600 2.4% (1/41), and ST10320 2.4% (1/41); Schwarzengrund, at 6% (37/616), was represented mostly by ST96 97.2% (36/37) and ST241 2.7% (1/37); Braenderup, at 5.8% (36/616), was largely represented by ST22 97.2% (35/36) and ST241 2.7% (1/36); and Typhimurium, at 5.6% (35/606), was associated primarily with ST19 85.7% (30/35), followed by ST213 8.5% (3/35), ST34 2.8% (1/35), and ST302 2.8% (1/35).

These seven serovars collectively represented 73.5% (453/616) of the genomes associated with more than one sequence type, illustrating a pattern where each serovar predominantly correlated with a primary ST. This primary ST consistently exhibited the highest frequency, representing the dominant lineage within each serovar and contributing a substantial percentage of its occurrences (Fig 2).

**Fig 3:**
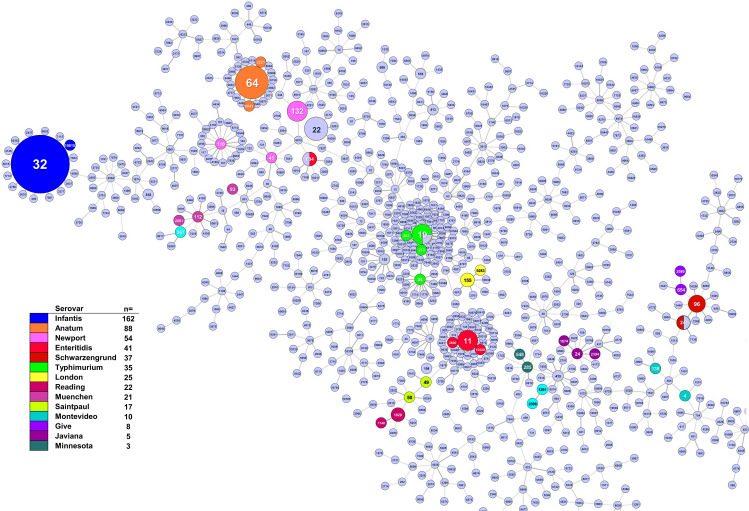
Minimum spanning tree illustrates relationships between sequence types and serovars of *S. enterica.* The diagram maps genetic relatedness based on shared sequence variants, highlighting both dominant STs and the genetic diversity within serovars.

To further confirm the close correlation between *S. enterica* serovars and ST, a goeBURST analysis was performed using ST designations by the Achtman scheme. The analysis highlights a strong correlation between specific serovars and their predominant ST. For the most frequent serovars, multiple STs were identified, with a clear predominance of a single ST in each case. However, in most instances, the less common STs were also genetically related to the main ST, as evidenced by the goeBURST analysis. For example, the serovar Infantis, ST10815 (n = 1) was a single-locus variant (SLV) of the predominant ST32 (n = 161). In Anatum, ST3221 (n = 4) and ST8041 (n = 1) were SLVs of the main ST64 (n = 83). Similar relationships were observed in other serovars: In Enteritidis, ST3600 (n = 1) and ST10320 (n = 1) were SLVs of ST11 (n = 38); in Schwarzengrund, ST241 (n = 1) was an SLV of the main ST96 (n = 36). In Typhimurium, ST34 (n = 1), ST213 (n = 3), and ST302 (n = 1) were SLVs of the main ST19 (n = 30) (Fig 3). In some cases, however, no close relationship was observed between the STs associated with a specific serovar, such as Newport, where ST132 (n = 43), ST118 (n = 10), and ST45 (n = 1) were separated by more than a triple-locus variant (TLV) distance (Fig 3).

### Antimicrobial resistance gene profile by serovar

**Fig 4:**
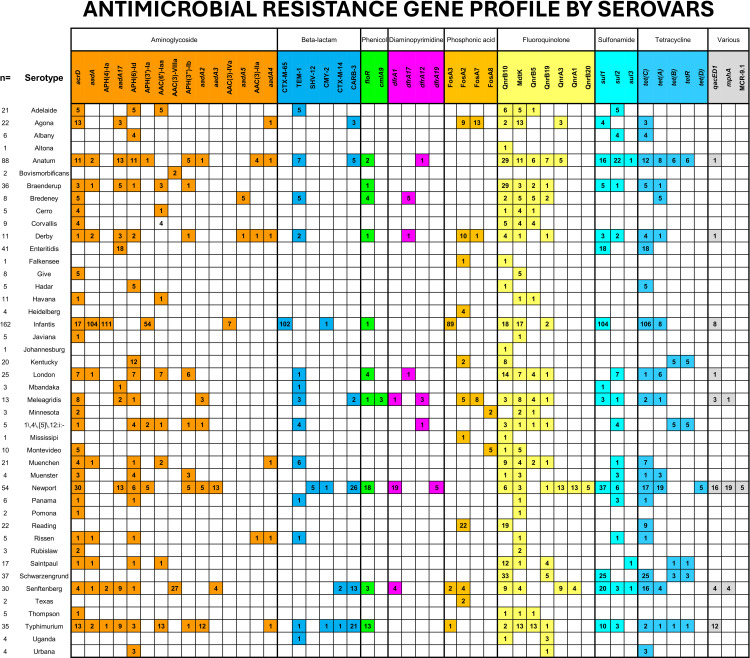
Antimicrobial resistance gene profile of *S. enterica* serovars. Resistance genes associated with aminoglycosides, β-lactams, fluoroquinolones, sulfonamides, and tetracyclines across major serovars. The numbers within the squares indicate the number of genomes carrying specific AMR genes.

The AMR gene profile across different *S. enterica* serovars reveals the presence of resistance determinants against multiple antibiotic classes, including aminoglycosides, β-lactams, phenicols, diaminopyrimidines, phosphonic acids, fluoroquinolones, sulfonamides, and tetracyclines. Among the most prevalent serovars, such as Infantis, Anatum, Newport, Enteritidis, Schwarzengrund and Typhimurium exhibits a complex AMR profile with multiple resistance genes spanning several antibiotic classes (Fig 4).

The Infantis serovar (n = 161), approximately 60% exhibit a broad resistance gene profile, prominently featuring aminoglycoside resistance genes such as *aph(4)-Ia*, *aadA*, and *aph(3´)-Ib*. This serovar also contains β-lactam resistance genes like *blaCTX-M-65* and shows resistance genes to fosfomycin and fluoroquinolones mediated by genes such as *fosA3* and *qnrB19*. Additionally, *sul1* and *tetA* genes related to sulfonamide and tetracycline resistance, respectively. The presence of multiple resistance determinants positions Infantis as a serovar with significant MDR potential.

The Anatum serovar (n = 88), exhibits a broad spectrum of resistance profiles. Common aminoglycoside resistance genes include *acrD*, *aadA17*, *aph(6)-Id*, *aac(3)-IId* and *aac(3)-VIIIa* and β-lactam resistance genes like *blaTEM-1* and *blaCARB-3*. Additionally, this serovar shows resistance genes to fluoroquinolones (*qnrA3*, *qnrB5*, *qnrB10*, *qnrB19*, and *mdtK)*, sulfonamides (*sul1*, *sul2* and *sul3*), and tetracyclines (*tetA*, *tetB tetC* and *tetR*). The extensive range of AMR genes in Anatum highlights its adaptability and potential resistance across various antibiotic classes.

Newport (n = 54), exhibits a broad spectrum of resistance profiles prominently featuring aminoglycoside resistance genes such as *acrD*, *aadA17*, *aph(6)-Id*, *aph(3´)-Ia*, *aadA2* and *aadA3* and β-lactam resistance genes like *blaSHV2* and *blaCARB-3*. Resistance to Phenicol and Diaminopyrimidine mediated by genes *floR* and *drfA1* respectively. Additionally, this serovar shows the presence of resistance genes to fluoroquinolones (*qnrA1*, *qnrB3*, *qnrB10*, *qnrB20*, and *mdtK)*, sulfonamides (*sul1*, *sul2* and *sul3*), and tetracyclines (*tetA*, *tetC* and *tetD*). *A*lso demonstrates a concerning resistance gene profile harboring *qacEdelta1*, facilitating antiseptic resistance. Resistance to macrolides, mediated by *mphA*, and *MCR-9.1* colistin resistance.

In contrast, Enteritidis (n = 41) exhibits a moderate AMR profile. Key resistance genes include those for aminoglycosides (*aadA17*), sulfonamides (*sul1*), and tetracyclines (*tetA*), while β-lactam and fluoroquinolone resistance determinants are notably absent. Although not as extensive as other serovars, the resistance profile of Enteritidis against multiple antibiotic classes remains a concern for treatment efficacy.

The Schwarzengrund serovar (n = 37) exhibits a limited AMR profile. The aminoglycoside and β-lactam resistance are notably absent. This serovar shows resistance to fluoroquinolones (*qnrB19*, *qnrB20*) and sulfonamides (*sul1*) and the presence of *tetB*, *tetC* and *tetR* genes indicates resistance to tetracyclines. While Schwarzengrund’s multidrug resistance is limited, its specific antimicrobial profile makes it a notable serovar in terms of AMR.

In Typhimurium (n = 35), a wide range of AMR genes were observed. Aminoglycoside resistance genes include *acrD*, *aadA*, *aadA17*, *aph(6)-Id*, *aac(6´)-Iaa* and *aac(3´)-lb* and *aadA2* were detected. β-lactam resistance is conferred by *blaTEM-1*, *blaCMY-2* and *blaCARB-3*, these genes were present in this serovar. Additionally, this serovar exhibits resistance to fluoroquinolones through (*qnrB5*, *qnrB10*, *qnrB19*, and *mdtK)*, sulfonamides (*sul1* and *sul2*), and tetracyclines (*tetA*, *tetB tetC* and *tetR*). The diverse AMR profile of Typhimurium indicates significant MDR potential across antibiotic classes.

### Phylogenetic analysis of *Salmonella* serovars and sequence types

The phylogenetic relationships among 818 *S. enterica* genomes and their correlation between the serovars and STs provide a comprehensive knowledge of the genetic structure in *S. enterica* population.

**Fig 5:**
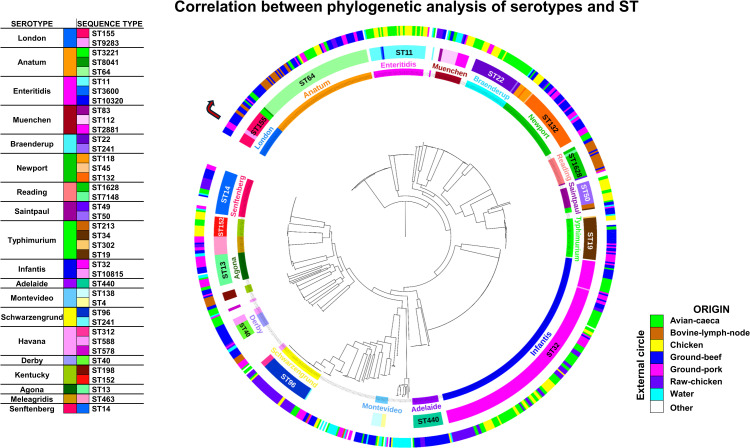
Correlation between phylogenetic analysis, serovars, and sequence types of *S. enterica.* This circular phylogenetic tree highlights genetic clustering of serovars and their associated STs. Outer circles represent the origin of isolates, showcasing the diversity of contamination sources contributing to the genetic structure of *S. enterica* in Mexico.

Maximum likelihood displays the core phylogeny in the central structure. This circular arrangement reveals genetic clustering patterns that align with genomic characteristics for each serovar. The internal circle maps serovar classifications, where each serovar tends to form distinct clusters along the phylogenetic tree. This alignment indicates a strong genetic cohesion within each serovar, with most isolates from the same serovar grouping together on closely related phylogenetic branches. This clustering pattern underscores the genetic stability and differentiation of serovars within the broader *S. enterica* population (Fig 5).

The middle circle shows the distribution of STs according to the Achtman scheme, which further aligns with the phylogenetic structure and serovar distribution. For most serovars, a predominant ST is observed as previously describe, correlating with the serovar-specific branches in the phylogenetic tree. This consistency between STs and phylogeny suggests that specific ST are generally conserved within serovars, supporting the association between genetic lineage and serovar identity.

The most external circle represents the origins of each genome, including avian caeca, bovine lymph node, chicken, ground beef, ground pork, raw chicken, water, and other sources. Unlike serovar and ST, the origins of the isolates do not exhibit a clear relationship with the phylogeny, or with the clusters defined by serovars and STs. Genomes from diverse origins are distributed across various phylogenetic branches, without a specific clustering pattern. This distribution suggests that *S. enterica* isolates with similar genetic profiles and serovar or ST classifications may arise from a variety of sources, highlighting the genetic similarity of isolates despite differing environmental or foodborne origins.

## Discussion

The genomic analysis of *S. enterica* conducted in this study contributes significantly to the overall understanding of *Salmonella* research in Mexico. It offers essential insights into contamination sources, sequence type and serovar diversity, antibiotic resistance gene profiles, and the close correlations observed between specific serovars and STs. This study highlights the importance of combining genomic and epidemiological approaches to provide a comprehensive comprehension of *Salmonella* dynamics.

This research contributes to the comprehension of the complex concerns associated with *Salmonella* infections and their public health implications by comparing our findings with previous studies [12,23–25]. The diversity of serovars identified in this study show the ecological and epidemiological complexity of *Salmonella* in Mexico. Consistent with previous findings, Infantis, Anatum, and Newport emerged as dominant serovars. The Infantis was closely associated with ST32, while Anatum was associated with ST64, reflecting a strong correlation between serovar and ST. These relationships show the evolutionary stability of specific serovars within environmental and their epidemiological contexts.

Notably, Newport exhibited significant genetic heterogeneity, suggesting constant genetic exchange and adaptation. This diversity may reflect its presence within a wide range of contamination sources, including beef, poultry, and vegetables. Such adaptability highlights the potential of this serovar to persist and spread within food production systems.

The identification of different serovars, even at lower frequencies, indicate the broad spectrum of *Salmonella* present in Mexico. The detection of Typhimurium, a serovar associated with global outbreaks, combined with regionally common serovars, suggests the simultaneous presence of both endemic and internationally disseminated strains. This finding also correlated with previous reports describing the predominance of specific serovars, including Newport and Typhimurium, which belong to distinct ST and highlight evolutionary stability and adaptability across diverse ecological niches [25].

Moreover, many of the serovars identified in this study showed a correlation with a single ST and were often associated to a low frequency of isolates. This observation is not exclusive to the Mexican context. Globally, several *S. enterica* serovars including Agona (ST13), Senftenberg (ST14), and Derby (ST40) have also been reported to exhibit low ST diversity, suggesting the predominance of stable clonal lineages across geographically distinct regions [26–28]. Such clonality may result from ecological specialization, and the successful expansion of clones through specific foodborne or environmental pathways. These trends underscore the evolutionary stability of certain serovars and emphasize the need for high-resolution genomic surveillance to track both established and emerging *Salmonella* lineages.

The temporal distribution of the main serovars Infantis, Anatum, and Newport shown a considerable heterogeneity during the collection period. While certain years showed higher number of isolates for specific serovars, such as Infantis in 2019, Anatum in 2021, and Newport in 2021–2022, these higher numbers were not restricted to a single collection event or a limited timeframe.

For instance, the 105 Infantis genomes collected in 2019 were distributed through 45 different collection events, suggesting a recurrent circulation rather than a single clonal expansion. Similar observations were made with the 28 Anatum isolates collected in 2021 throughout 9 distinct dates, and the 27 Newport isolates collected during 9 different events in 2022. The diversity in collection dates suggests that the observed temporal dispersion is more likely attributable to constant prevalence rather than the emergence of a specific clonal lineage, indicating temporal dispersion most than a localized or temporary outbreaks.

The problem of antimicrobial resistance remains a critical area of study in *Salmonella* research, and the findings of this study emphasize the urgent necessity of dealing with this issue. A wide range of antimicrobial gene profiles was observed, particularly in the serovars Infantis, Anatum, Newport, and Typhimurium, with a significant presence of resistance genes against aminoglycosides, fluoroquinolones, sulfonamides, and tetracyclines.

Notably, in Infantis many of these AMR genes, including *tet*, *sul*, *aadA*, *blaCTX-M-65* and *fosA3*, are consistent with those commonly associated with the pESI plasmid, a known driver of multidrug resistance in this serovar [29].

The detection of genes such as *mphA* and *MCR* in Newport further highlights the genetic mechanisms underlying their resistance capabilities. These findings align with previous reports indicating that Newport frequently harbors genetic determinants conferring resistance to multiple antibiotic classes. Moreover, the observed resistance patterns are consistent with global trends, particularly in regions with intensive agricultural practices, as noted by Gómez-Baltazar et al. [25]. Their study also emphasized the role of mobile genetic elements in the dissemination of resistance genes, a mechanism that facilitates the rapid adaptation of *Salmonella* serovars to antimicrobial pressures. This dynamic is crucial for understanding the persistence of MDR strains across various ecological and geographic contexts.

Comparative genomic analyses revealed substantial concordance between phenotypic resistance profiles and genotypic data. This consistency underscores the efficacy of whole genome sequencing (WGS) in monitoring antimicrobial resistance, enabling the reliable identification of resistance elements and facilitating real-time observation of resistance patterns.

The results of this study have substantial implications for public health and food safety in Mexico. Animal-derived food products, especially beef and chicken, were recognized as significant reservoirs of MDR *Salmonella*. The significant prevalence of MDR strains, maintained by their persistence in food production and distribution networks, underscores the urgent necessity for improved control techniques. The association between specific serovars and sequence types offers significant insights into the pathways of evolution of *S. enterica*.

In *S.* Infantis, genetic stability suggests elevated potential for prolonged persistence within specific ecological niches. In contrast, the genetic heterogeneity observed in *S.* Newport reflects its evolutionary dynamism, which may enhance its adaptability to diverse environmental pressures.

These findings underscore the necessity of mitigating contamination at different points of the food supply chain. A comprehensive approach is necessary to limit the risk of *Salmonella* contamination and transmission, including farm-level treatments such enhanced biosecurity measures, as well as post-harvest processing and distribution.

## Conclusion

This study provides detailed insights into the epidemiology of *S. enterica* in Mexico, emphasizing its genetic diversity, prevalence of common serovars, identification of main sequence types, and prevalence of antimicrobial resistance genes

*S* Infantis, the most common identified serovar, reflects findings from other regions where it is predominantly associated with Foodborne and livestock-associated sources. This serovars exhibits a wide antimicrobial resistance gene profile to multiple antimicrobial classes. Other dominant serovars, such as Infantis, Newport, and Typhimurium, also show strong associations with MDR gene profiles, emphasizing the urgent need for antimicrobial stewardship and vigilant surveillance in food production systems.

The association of single serovars in *S. enterica* with multiple ST, suggest their genetic adaptability as key factor in their persistence and evolution under environmental and antibiotic selection pressures [18]. This genetic diversity rises from several mechanisms, including homologous recombination, horizontal gene transfer, and spontaneous mutations, all of them contribute to genetic diversity within the members of a single serovar [30]. The presence of multiple ST within a single serovar reflects genomic plasticity, allowing adaptation to a wide range of ecological niches. This plasticity is particularly important for the survival of *S. enterica* in diverse environments, including food production systems, animal reservoirs, and clinical settings [31]. However, a predominant ST was consistently observed among serovars associated with multiple STs, suggesting a strong correlation between each serovar and a dominant ST in most cases analyzed in this study.

Furthermore, the genetic heterogeneity within a serovar has implications in the AMR evolution. The acquisition and dissemination of resistance genes via mobile genetic elements, such as plasmids and integrons, will be facilitated by genetic recombination among different lineages within the same serovar [30,32].

From an epidemiological perspective, the presence of multiple STs within a serovar complicates outbreak investigations and source attribution, as serovar-based typing alone does not necessarily correlate with phylogenetic lineage or clonal expansion [18]. This highlights the importance of high-resolution genomic approaches, such as core genome multilocus sequence typing and whole-genome sequencing, to accurately delineate transmission pathways and evolutionary trajectories of *S. enterica* [18].

This complexity complicates containment strategies, need an integrated approach that considers serovar and sequence type variability. For instance, reducing antibiotic use in agriculture, particularly in poultry production, could mitigate the spread of MDR strains like Infantis.

Globally, *S. enterica* remains a leading cause of foodborne illness, with millions of cases annually and an escalating threat from MDR strains that compromise treatment efficacy. The rising occurrence of MDR *Salmonella* in low- and middle-income countries, such as Mexico, highlights the ongoing deficiencies in mitigation strategies. This study highlights the necessity for strong monitoring systems, especially within poultry supply chains, which can improve the early identification of emerging MDR strains and inform targeted interventions.

The findings carry substantial implications, given that MDR *Salmonella* strains have the potential to disseminate across borders via trade and travel. Collaborating internationally on AMR surveillance and sharing data, as advised by the WHO, is crucial for tackling this cross-border challenge. Regions such as Mexico can enhance their public health defenses against MDR *Salmonella* and control foodborne pathogens by implementing surveillance and strengthening international cooperation. A multifaceted approach is necessary to address these challenges which include reducing antibiotic use in agriculture, the enhancement of monitoring of S. *enterica* Foodborne and livestock-associated sources, and the adoption of international collaboration to mitigate the public health problem presented by MDR *Salmonella*.

## Supporting information

S1 Metadata Genomic metadata of 818 *S. enterica* isolates from Mexico. (XLSX)

S2 Metadata ANI identity from 818 *S. enterica* genomes. (XLSX)
